# Production and Partial Characterization of Bioactive Compounds from Underutilized Marine Bioresources for a Cosmetic Formulation: Cytotoxicity and Bioactivity Evaluation

**DOI:** 10.3390/ijms242015380

**Published:** 2023-10-19

**Authors:** María Blanco, Ana C. Sánchez, Begoña Correa, José Antonio Vázquez, Andrea Vázquez, Ricardo I. Pérez-Martín, Carmen G. Sotelo

**Affiliations:** 1 Grupo de Bioquímica de Alimentos, Instituto de Investigaciones Marinas, Consejo Superior de Investigaciones Científicas, Eduardo Cabello, 6, 36208 Vigo, Spain; asanchez@iim.csic.es (A.C.S.); begonacorrea@iim.csic.es (B.C.); ricardo@iim.csic.es (R.I.P.-M.); carmen@iim.csic.es (C.G.S.); 2 Group of Recycling and Valorisation of Waste Materials (REVAL), Instituto de Investigaciones Marinas, Consejo Superior de Investigaciones Científicas, Eduardo Cabello, 6, 36208 Vigo, Spain; jvazquez@iim.csic.es; 3 Iuvenor Lab, Poligono Industrial de Uceira, Vial Uno, Parc 11, 32500 Ourense, Spain; andrea.vazquez@iuvenor.es

**Keywords:** circular economy, fish by-product, hydrolyzed collagen, hyaluronic acid, pro-collagen I, mRNA expression

## Abstract

Hydrolyzed collagen, glycogen, and hyaluronic acid, obtained through the biotechnological valorization of underutilized marine bioresources, fulfill cosmetic industry requirements for sustainable products produced under circular economy principles. Hydrolyzed collagen was obtained by hydrolyzing blue shark collagen with papain and ultrafiltration. Glycogen was isolated from industrial mussel cooking wastewaters through ultrafiltration, precipitation, and selective polysaccharide separation. Hyaluronic acid was produced by fermentation, purification, and depolymerization. The main objective was to test the feasibility of including these three biomolecules in a cosmetic formulation as bioactive compounds. For this, the in vitro irritant potential of the three ingredients and also that of the cosmetic formulation was assayed according to the Reconstituted Human Epithelium Test method OECD 439. Moreover, an in vitro assessment of the effect of hydrolyzed collagen and hyaluronic acid combinations on mRNA expression and collagen type I synthesis was evaluated in adult human fibroblasts. This study establishes, for the first time, the potential use of particular hydrolyzed collagen and hyaluronic acid combinations as stimulators of collagen I synthesis in fibroblast cultures. Besides, it provide safety information regarding potential use of those biomolecules in the formulation of a cosmetic preparation positively concluding that both, ingredients and cosmetic preparation, resulted not irritant for skin following an international validated reference method.

## 1. Introduction

Nowadays, there is a growing social concern and awareness for social and environmental issues [1,2]. The cosmetic industry, not avoiding the challenge, is working to meet the requirements of consumers’ preferences, demanding natural and sustainable products with a low environmental impact and producing under circular economy principles [3]. Marine-derived ingredients, which are considered safe, nutritious, and have several remarkable properties (antioxidant, moisturizing, or anti-aging effects) [4,5], are excellent candidates to be incorporated into cosmetic formulations, avoiding the use of mammal-derived sources, which are considered less eco-friendly and possess more restrictions on their use (religious or disease concerns). As a result, the cosmetic industry is increasingly incorporating a wide variety of marine-origin ingredients in their formulations with different functionalities, claiming “marine” as a distinctive marketing signal [4,6,7]. Some marine ingredients are already being used by different cosmetic brands; however, the information regarding the sustainability of their sources and production practices has not been considered an important aspect to be addressed for decades [6]. The cosmetic industry might tackle this challenge, assuming circular economy principles regarding the source of its ingredients. The circular economy in fisheries and aquaculture industry sectors implies an efficient use of all side streams and by-products generated in a particular industry, using biotechnology and physicochemical procedures to transform them into valuable bio-compounds useful for a wide variety of pharmaceutical, food/feed, and medical applications.

The fish processing industry generates thousands of tons of by-products (heads, skins, viscera, trimmings, protein-containing residuals, etc.) yearly, during the transformation of fish or seafood [8,9], which contain valuable biocomponents (lipids, proteins, enzymes, or mineral fractions) [10]. If those by-products are not adequately managed or valorized, it leads to significant economic loss, pollution, and a waste of natural resources. Fish and seafood by-products therefore represent an important factor in contributing to a more sustainable exploitation of marine resources. The valorization of fish industry by-products into high-value-added products will contribute to income diversification, economic sustainability, and resilience in the fishing industrial sector. Valorization through the biotechnological and chemical transformation of fish-filleting skin by-products, mussel-processing wastes, and tuna viscera by-products to obtain hydrolyzed collagen (HC), glycogen (G), and hyaluronic acid (HA), respectively, has been studied previously [11,12,13,14,15]. These three biomolecules obtained through the valorization of fishery by-products are regarded as environmentally friendly or sustainable ingredients that are ideal to fulfill social, cultural, or industrial eco-sustainable requirements and are of particular interest to the cosmetic industry as they possess adequate bioactivities.

Marine hydrolyzed collagen has been reported to provide a valuable source of peptides with many bio-functional properties, including antioxidant, cell proliferation effects on human dermal fibroblast, wound-healing activities, skin hydration/elasticity capacity, or anti-wrinkle activity [14,16,17]. Glycogen has been included in cosmetic formulations, principally as an emollient and velvety agent. Hyaluronic acid has been previously reported to exhibit remarkable skin regeneration and collagen-stimulating efficacy [18]. Furthermore, the combination of collagen and hyaluronic acid has advantages over the use of both ingredients separately for tissue engineering and cosmetic applications by stimulating chondrocyte and fibroblast growth in vitro [19].

The main purpose of this study Is to evaluate the combination of these three biomolecules obtained from marine by-products as ingredients for cosmetic products. The study has developed the following: (i) an analysis of the irritant potential of the ingredients; (ii) an analysis of the irritant potential of cosmetic formulation; (iii) an in vitro assessment effect of the hydrolyzed collagen and the hyaluronic acid combinations on RNA expression; and (iv) in vitro assessment effect of the hydrolyzed collagen and the hyaluronic acid combinations on collagen type I synthesis in adult human fibroblasts. As far as the authors’ knowledge is concerned, this is the first attempt to study these marine ingredient combinations for cosmetic products.

## 2. Results

### 2.1. In Vitro Skin Irritation Assessment of Bioactive Compound Ingredients

**Figure 1:**
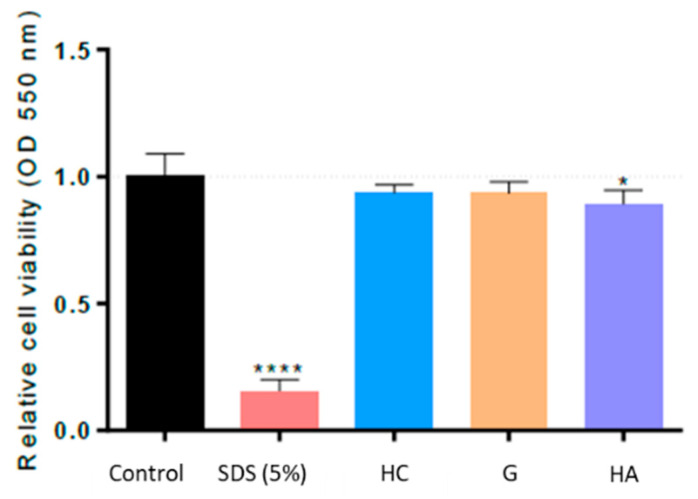
Cell viability in Reconstructed Human Epidermis, normalized to non-treated control, after treatment for 60 min with positive control SDS at 5% (*w*/*v*) and HC, G, and HA at 2% concentration. * represents statistical significance with *p* value < 0.05. **** represents statistical significance with *p* value < 0.0001.

According to EU classification, the irritancy potential of test substances is used to distinguish between skin-irritating and non-skin-irritating test compounds [20,21]. The irritancy potential of bioactives (HC, G, and HA) was predicted by means of the cell viability of tissues exposed to them following the OECD 439 protocol [22]. According to this protocol, a test substance is considered an irritant to the skin if the mean relative viability after treatment incubation is less than or equal to 50% of that of the negative control. The concentration of bioactives was assayed at 2% (*w*/*v*) according to the OECD 439 protocol [22]. Following this protocol, the cell viability results indicated that the treatment with HC and G at 2% concentration did not show any significant decrease in cell viability when topically applied during 1 h in RHE Model EpiDermTM, compared to the untreated control (Figure 1). On the other hand, the treatment with HA at 2% concentration decreased cell viability by only 11.0 ± 4.7% compared to the untreated control. In conclusion, based on these results and according to the OECD 439 protocol [22], the tested three ingredients can be considered non-irritants for the skin.

### 2.2. Viability of Fibroblast Cell Cultures Treated with HC and HA Combinations

The cell viability in human dermal fibroblast was used to perform a preliminary screening on the effects of treatments on fibroblast and to select the most appropriate HC and HA combinations to perform further experiments. So, the effect of HC and HA on fibroblast cell viability was tested using 25 different combinations of HC and HA concentrations (Table 1) after 24 h of incubation. These combinations were selected based on previous studies [13,23,24]. The results showed that approximately 97% of the total variation is explained by the treatments (Anova Welch, F(24, 45.12) = 61.63; *p* < 0.01; η^2^ = 0.97), meaning that treatments affect cell viability. When comparing the performance of the different combinations, the highest viability was found with combination 16 (Table 1). No significant differences (*p* > 0.05) were found between treatment 16 and treatments 6, 7, 8, 11, 12, 13, 17, 21, 22 and 23 (Table 1).

**Figure 2:**
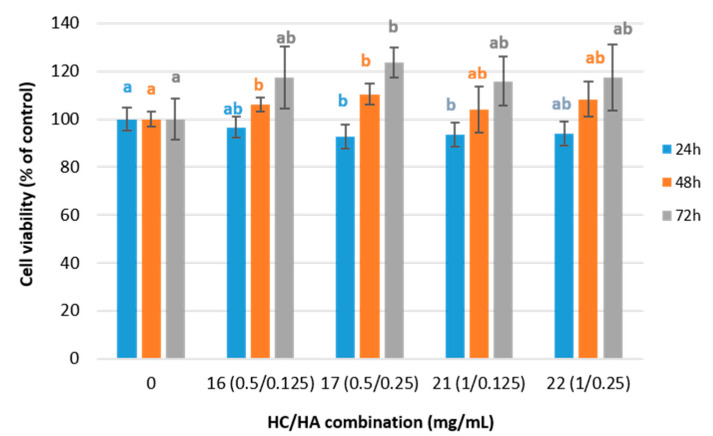
Cell viability values (%) for the fibroblast cell cultures treated with HC and HA combinations and normalized to the control group (0 treatment, Table 1) after 24, 48, and 72 h of incubation. Values are shown as means with standard deviation. Different letters indicate significant differences between HC/AH combinations (*p* < 0.05).

The combinations of 0.5 or 1 mg/mL HC with 0.125 or 0.25 mg/mL HA corresponding to treatments 16, 17, 21, and 22 were selected for further viability test. The results showed that cell viability after 24 h of incubation was only significantly lower than the control in fibroblast cell cultures treated with 17 and 21 treatments (Anova, F(5, 54) = 44.471; *p* < 0.01, η^2^ = 0.82) (Figure 2). However, in all cases, the percentage of viability remains above the threshold of 70%, which is considered the limit for cell cytotoxicity [25]. After 48 h of incubation (Anova Welch, F(4, 12.09) = 5.367, *p* < 0.05, η^2^ = 0.64, post-hoc test Games–Howell), cell viability increased significantly with treatments 16 and 17, and only in treatment number 17 did it increase significantly and progressively up to 72 h, reaching a cell viability percentage of 123.5 ± 6.3% (Anova, F(4, 29) = 4.00, *p* < 0.05, η^2^ = 0.39).

### 2.3. Effect of HC and HA Combination on mRNA Pro-Collagen I Expression of Fibroblast Culture

**Figure 3:**
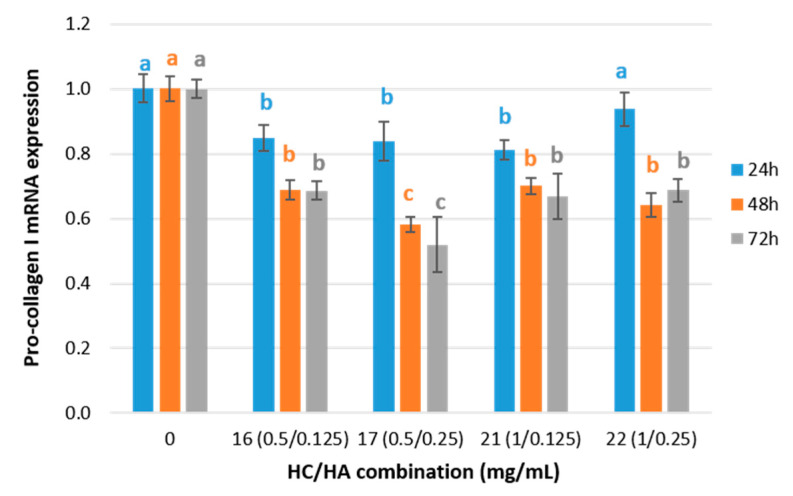
Pro-collagen I mRNA expression for the fibroblast cell cultures treated with selected HC/HA combinations at 24, 48, and 72 h and normalized to the control group. Values are shown as means with standard deviation. Different letters indicate significant differences between concentration groups (*p* < 0.05).

Collagen expression was investigated at the gene level by determining mRNA expression of pro-collagen I gene (COL_I) in a fibroblast cell culture incubated with the four selected treatments of HC/HA and control (Table 1 and Figure 3) for 24, 48, and 72 h.

Pro-collagen I mRNA expression of treated cell culture decreased significantly (17 ± 2%) after 24 h of incubation with treatments 16, 17, and 21 (Anova, F(5, 53) = 28.00, *p* < 0.01, η^2^ = 0.75; Scheffé post-hoc test). However, after 48 h, there was an overexpression that increased up to 30 ± 1% with the treatments 16 and 21 and up to 52% with the combination number 17 (Anova, F(4, 29) = 155.58, *p* < 0.01, η^2^ = 0.961, test post-hoc Scheffé) remaining without significant differences after 72 h of incubation (t-Student test for all combinations HC/HA concentrations, *p* > 0.05), (Anova, F(4, 29) = 62.75, *p* < 0.01, η^2^ = 0.91, Scheffé post-hoc test).

No significant correlation between pro-collagen I mRNA expression and cell viability was observed after 24 h of incubation (r24 h = 0.336, *p* > 0.01). Moreover, after 48 h and 72 h of incubation, a significant moderate inverse correlation between cell viability and pro-collagen I mRNA expression was appreciated (r48 h = −0.633 **; r72 h = −0.635 **). That is, as the incubation time increases, it also increases cell viability and decreases pro-collagen I mRNA expression. Similar results were observed by other authors [26,27,28] when human dermal fibroblasts were treated with high-molecular-weight (Mw) HA.

### 2.4. The Effect of HC and HA Combinations on Pro-Collagen I Synthesis (ELISA) of Fibroblast Culture

**Figure 4:**
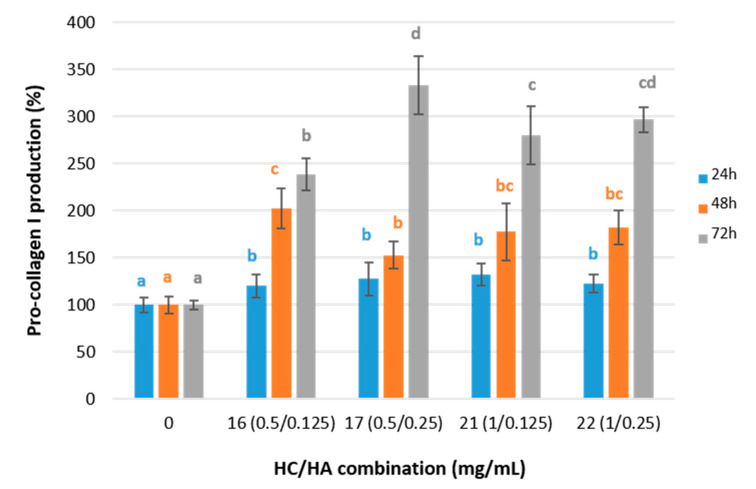
Pro-collagen I production for the fibroblast cell cultures treated with HC and HA at 24, 48, and 72 h and normalized to the control group. Values are shown as means with standard deviation. Different letters indicate significant differences between treatments (*p* < 0.05).

When the pro-collagen I synthesis was determined in adult human dermal fibroblasts treated with any of the HC/HA combinations, a 25.7 ± 5.3% increase was observed after 24 h of incubation (test Anova, F (5, 59) = 17.01, *p* < 0.01 η^2^ = 0.61, Scheffé post-hoc test) (Figure 4). Such overexpression increased significantly after 48 h of incubation, especially for the fibroblasts treated with treatment 16, which reached 202.4 ± 21.2%, a 2-fold increase compared to that of untreated cells (test Anova, F (4, 29) = 23.54, *p* < 0.01 η^2^ = 0.79). Finally, after 72 h of incubation, all the combinations led to a prominent overexpression of pro-collagen (I, especially treatment 17, which tripled (332.9 ± 30.7%) that of untreated cells test Anova, F (4, 26) = 320.66, *p* < 0.01, η^2^ = 0.98).

Moreover, the correlation between pro-collagen I production and cell viability was moderately negative (r24 h = −0.567 **) after 24 h of incubation. Although a non-significant correlation was observed after 48 h of incubation (r48 h = −0.348, *p* > 0.01), the correlation was found to be positive after 72 h of incubation (r72 h = 0.597 **). When analyzing the correlation between pro-collagen I mRNA expression and pro-collagen I production, a moderately negative trend was observed after 24 h and 48 h of incubation (r24 h = −0.473 **, r48 h = −0.652 **), which became a very strong negative correlation after 72 h (r = −0.934 **). Finally, no increase in viability, mRNA expression, or pro-collagen I production was observed for periods shorter than 24 h (Figure S1, Supplementary Material).

### 2.5. The Effect of HA on the Expression and Synthesis of Pro-Collagen I in Fibroblast

Based on the stimulatory effect of HC on the synthesis of pro-collagen I in fibroblast cultures results previously reported in Sánchez et al. [13], the effect of a particular combination of a fixed HC concentration with different concentrations of HA was evaluated in this study. The production of pro-collagen I increased by 1.3- and 1.5-fold in treatments 17 and 22, respectively, compared to the fibroblast cultured with only 0.5 mg/mL and 1 mg/mL of added HC (Figure S2a, Supplementary Material). Therefore, the percentage of the total variance that is explained by the interaction between HC and HA increases from 6.1% after 24 h of incubation up to 22.9% after 48 h and up to 10.4% after 72 h of incubation (two-way ANOVA, *p* < 0.01, η^2^_24_ h = 0.061, η^2^_48 h_ = 0.229, η^2^_72_ h = 0.104). Nevertheless, the opposite effect was observed for the pro-collagen mRNA expression, which undergoes a significant downregulation effect when HA is added to the fibroblast cell cultures (Figure S2b, Supplementary Materials). That is, when the HC concentration was fixed at 0.5 mg/mL, a 30% Pro-collagen I mRNA downregulation effect was observed after 24 h of incubation for the two HA concentrations tested (0.125 and 0.250 mg/mL). After 48 and 72 h of incubation, significant differences between both HA concentrations were observed, showing a pro-collagen I mRNA downregulation of 36% and 55% for 0.125 and 0.250 HA concentrations, respectively (48 h: Anova Welch, F(2, 9.157) = 101.521, *p* < 0.001, η^2^ = 0.96, post-hoc Games–Howell; 72 h: Anova Welch, F(2, 7.471) = 36.92, *p* < 0.001, η^2^ = 0.91, post-hoc Games–Howell). When the HC concentration was fixed at 1 mg/mL, the greatest gene downregulation (46%) occurred after 24 h of incubation with the lowest HA concentration (0.125 mg/mL), and 38% downregulation occurred with the highest HA concentration (0.25 mg/mL) (Anova Welch, F(2, 10.851) = 103.504, *p* < 0.001, η^2^ = 0.95, post-hoc Games–Howell).

Regarding pro-collagen I production, when the HC concentration was set at 0.5 mg/mL, the highest production (20% higher) was obtained at 24 h of incubation when HA was absent (Anova, F(2, 27) = 9.485, *p* < 0.001, η^2^ = 0.413, post-hoc Scheffé); at 48 h, the presence or absence of HA did not lead to significant differences in the pro-collagen I production between the HC/HA combinations with a fixed HC concentration at 0.5 mg/mL (Anova, F(2, 15) = 6.440, *p* < 0.05, η^2^ = 0.462, post-hoc Scheffé). However, at 72 h, combination 17(0.5/0.25) tripled the control production and was 30% higher than the production achieved with combinations 15 (0.5/0) or 16 (0.5/0.125) (Anova, F(2, 13) = 44.871, *p* < 0.05, η2 = 0.873, post-hoc Scheffé). When the HC concentration increased up to 1 mg/mL, the greatest pro-collagen I production was observed at 24 and 48 h of incubation when the HA concentration was 0 mg/mL (34% and 21% higher, respectively) (Anova 24 h, F(2, 27) = 101.690, *p* < 0.001, η^2^ = 0.883, post-hoc Scheffé; Anova 48 h, F(2, 15) = 6.792, *p* < 0.05, η^2^ = 0.475, post-hoc Scheffé). The trend was reversed after 72 h of incubation with an increment of 19% (Anova Welch 72 h, F(2, 7.706) = 39.6, *p*< 0.01, η^2^ = 0.91, post-hoc Games–Howell).

### 2.6. In Vitro Skin Irritation Assessment of Cosmetic Preparation according to the RHE Test Method, OECD 439

**Figure 5:**
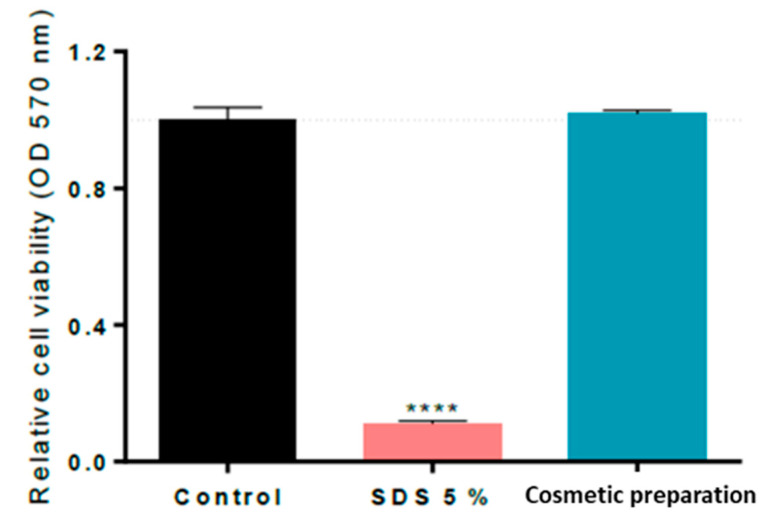
Cell viability in Reconstructed Human Epidermis (RHE), normalized to control (RHE without applying the cosmetic preparation), after treatment for 60 min with positive control SDS at 5% or cosmetic preparation at 100% concentration. **** represents statistical significance with *p*-value < 0.0001.

Cell viability quantification through MTT assay results indicated that treatment with the cosmetic preparation at a 100% concentration did not show any significant decrease in cell viability when topically applied during 1 h in the RHE Model EpiDermTM compared to the untreated control (cells without the cosmetic preparation), whereas the positive control (SDS at 5% (*w*/*v*)) significantly decreased its cell viability by 89%, as shown in Figure 5. The percentage of HC, G, and HA in the final cosmetic formulation (0.4%, 0.15%, and 0.5%, respectively) could be increased by up to 2% because the results of the in vitro skin irritation assessment of individual ingredients confirmed that ingredients at 2% were not irritants for the skin.

## 3. Discussion

### 3.1. In Vitro Skin Irritation Assessment of Active Ingredients and Cosmetic Formulation

To verify that the topical application of active ingredients and cosmetic formulations does not cause irritation to the skin and that they can be safely provided to consumers, skin irritation testing becomes an important requisite for the preparation and safety of cosmetics. Since the European Commission prohibited animal experiments for cosmetics in 2013 in the European Union, it is necessary to use alternative methodologies for the analysis of the irritant potential of those ingredients/substances intended for cosmetic applications that were previously tested in animals. Skin irritation potential can be determined using in vitro systems, as long as they mimic the skin barrier. In this context, the Organization for Economic Co-operation and Development (OECD) guideline 439 [22] provides an internationally validated reference method that allows the identification of irritant chemicals using RHE models instead of live animals. To accomplish this with the international regulations, one of the aims of this work was to assess the cutaneous irritation potential of three ingredients (HC, G, and HA) and that of the cosmetic formulation prepared with those ingredients, according to the in vitro toxicological study established in the OECD 439 [22] guidelines, through cell survival quantification by MTT assay in keratinocytes. The MTT procedure is habitually performed to determine the effects produced by a substance or treatment upon cell viability, which may be interpreted as toxic effects (cytotoxicity) if cell viability is compromised or as stimulating effects (proliferation) if cell viability increases, comparing the treatments with the untreated control group [29,30]. Cell viability results indicated that the three tested ingredients and the cosmetic formulation were not irritants for human skin in the tested trial doses because, although HA significantly decreased cell viability by 11.0 ± 4.7% compared to the untreated control, the mean relative viability after treatment was higher than 50% of the negative control in all cases. These results are of high importance for the safety evaluation of the potential use of the cosmetic formulation prepared, and, as far as the authors know, this information has not been previously provided for these active ingredients obtained through the valorization of fish industry by-products.

### 3.2. The Effect of HC and HA Combinations on Fibroblast Viability, mRNA Pro-Collagen I Expression, and Pro-Collagen I Synthesis

The results revealed a stimulatory effect of HC/HA combinations on fibroblast metabolism, leading to both an increase in cell viability and pro-collagen I synthesis, without observing an increase in pro-collagen I mRNA expression. Similar results were also observed by other authors [26,27,28] when human dermal fibroblasts were treated with high-molecular-weight HA, and no change in pro-collagen I mRNA expression was observed in contrast to the increase in cell viability.

There is not just a single factor explaining these results. Thus, the amount of protein accumulated from a particular transcript might be influenced not only by the amount of mRNA present in the cytoplasm but also by the rate of translation of the mRNA and its stability [31]. Furthermore, the increased pro-collagen I synthesis displayed by combinations of HC and HA concentrations might also be linked to a particular HC amino acid profile and/or to its particular molecular weight profile, which could have an effect on cellular mechanisms or synthesis pathways [32,33,34]. Moreover, the individual or synergistic effects produced by the combination of HA and HC have been considered by comparing the present results with those obtained previously [11]. A synergistic effect might be responsible for the increase in pro-collagen I up to 1.3- and 1.5-fold when HC and HA were combined compared to the fibroblast treated only with HC [11].

It has been reported that HA can affect cell behavior and metabolism [35] since it is recognized by cell surface receptors such as CD44 (cluster differentiation 44) and RHAMM (receptor for HA-mediated motility), activating signaling pathways involved in the stimulation of fibroblast proliferation and collagen production [32,36,37] as well as other proteome alterations [38]. Moreover, HA polymers have different biological activities on cells, which seem to be driven by their molecular weight [28,37,39]. Previous studies have demonstrated that the modification of HA molecular weight could increase type I and III collagen expressions in dermal fibroblasts [26,28]. Low-molecular-weight HA has been reported to stimulate cell proliferation, whereas higher HA molecular weight fractions exert an inhibitory effect [37]. Our findings, employing a 51 kDa HA, are in line with those previous studies [38,40,41], suggesting that exogenous low-molecular-weight HA affects fibroblast proliferation and collagen synthesis.

Regarding pro-collagen I synthesis results, it is noteworthy that although the HA concentration varies, no significant differences were observed between treatments 21 and 22 with a 1 mg/mL HC concentration (Figure 4). On the other hand, maintaining the HC concentration constant at 0.5 mg/mL led to significant differences in the pro-collagen I synthesis between treatments 16 and 17, both at 48 h and 72 h. At 48 h, the pro-collagen synthesis was increased in treatment 16 (lowest HA concentration), while at 72 h, the increment was significantly higher in treatment 17 (higher HA concentration). It seems that the stimulating effect of HA on pro-collagen I synthesis in fibroblast cell cultures was hindered as the collagen concentration increased in the treatments. The significant effect of a higher HA concentration on the pro-collagen I synthesis at 0.5 mg/mL collagen concentration observed at 72 h is not a consequence of a higher mRNA expression as it has been observed as a downregulating effect (Figure 3). However, it might be explained by the cell viability results (Figure 2), which demonstrate significant cell viability increases in treatments 16 and 17. Edgar et al. [42] also observed increments of structural extracellular matrix proteins after the addition of collagen peptides together with other additives, including HA, in fibroblast cell cultures and explained those results with different factors, including cell viability and inhibition of metalloproteases.

## 4. Materials and Methods

### 4.1. Bioactive Compound Ingredients

The following ingredients, hydrolyzed collagen (34 kDa), glycogen (2551 kDa), and hyaluronic acid (51 kDa and 1500 kDa, respectively), were obtained, as indicated in [11,12,43,44], and refrigerated (−20 °C) until use for experiments and the preparation of the cosmetic formulation. Briefly, hydrolyzed collagen was obtained by first treating *Prionace glauca* skins with 10 volumes of 0.1 M NaOH and stirring in a cold room (4 °C) for 24 h. The liquid was discarded and the NaOH-treated skins washed until neutral pH. Washed skins were stirred for 24 h with 10 volumes of 0.5 M acetic acid. The extract was centrifuged (3000× *g*/15 min), and the supernatant was dialyzed against water using 14 kDa molecular weight cut-off cellulose membranes and freeze-dried. The obtained acid-soluble collagen was hydrolyzed in a controlled pH-Stat system (Metrohm, Herisau, Switzerland) using papain (Merck, KGAA, Darmstadt, Germany) (E/S: 1/20) for 30 min at 65 °C in a water bath and ultra-filtrated using 30 kDa and 10 kDa Mw cut-off Amicon Ultra Device membranes to achieve the desired molecular weight. Glycogen was isolated from industrial mussel cooking wastewaters after its concentration by ultrafiltration membrane with 100 kDa cut-off (spiral polyethersulfone, 0.56 m^2^, Prep/Scale-TFF, Millipore Corporation, USA), followed by protein precipitation by isoelectric point (pH 4.5) with 5 M HCl, a selective polysaccharide separation using alcoholic precipitation, and finally, an oven-drying step [43]. Hyaluronic acid was produced by fermentation of *Streptoccocus zooepidemicus* bacterium in low-cost nutritive medium, including tuna viscera peptone [12]. The high-molecular-weight HA (1500 kDa) obtained after exhaustive purification (enzyme proteolysis combined with chemical precipitation and ultrafiltration procedure) was depolymerized using 7 units of bovine testes hyaluronidase/mg of HA at 37 °C/pH4/200 rpm for 2 h of hydrolysis rendering of the 51 kDa HA [44]. Molecular weights of biopolymers were determined by gel permeation chromatography (GPC), according to previously published protocols [11,43,44]. GPC was performed using Agilent 1260 HPLC system consisting of quaternary pump (G1311B), injector (G1329B), column oven (G1316A), DAD (G1315C) refractive index (G1362A), and dual-angle static light-scattering (G7800A) detectors, along with specific Suprema and Proteema size exclusion columns (PSS, Mainz, Germany).

#### 4.1.1. In Vitro Skin Irritation Assessment of Ingredients according to RHE Test Method OECD 439

To perform the in vitro skin irritation test OECD 439 [22], a three-dimensional Reconstituted Human Epidermis (RHE) RHE Model EpiDermTM EPI-200-SIT was used. It consisted of a 0.63 cm2 tissue made of normal, human-derived epidermal keratinocytes, which were cultured to form a multi-layered, highly differentiated model on an inert polycarbonate filter at the air–liquid interface. A total of 30 μL of each ingredient (HA, G, and HC) was topically applied at 2% (*w*/*v*) concentration onto the surface of RHE for 60 min at 37 °C. The HA used was a mixture of low and high Mw (50%). At the end of the exposure period, tissues were rinsed with phosphate buffer solution (DPBS) and transferred to fresh medium for 24 h. Then, the medium was changed, and the cells were incubated for another 18 h. Cell viability quantification was evaluated using MTT assay [29]. Tissues were treated for 3 h at 37 °C with 0.3 mL of MTT solution, and then 2 mL of Isopropanol was added and left for 2 h at room temperature (RT) with gentle agitation for formazan extraction. Control was similarly prepared, without the addition of active ingredients. The concentration of formazan was measured by determining the absorbance at 570 nm on a scanning multiwell spectrophotometer (Halo Led 96, Dynamica Scientific Ltd., Livingston, UK).

Five replicates were used. Data were statistically analyzed using one-way ANOVA test with statistical significance set at *p* < 0.05. Results were expressed in terms of % cell viability relative to negative control (Isopropanol) using the following formula:

$$\%\;cell\;viability=\frac{Abs\;sample-Abs\;blank}{Abs\;negative\;control-Abs\;blank}\times 100$$

#### 4.1.2. Viability Assay of Hydrolyzed Collagen and Hyaluronic Acid Combinations in Fibroblast Cells

For the treatment of cultured cells with HC and HA combinations, adult human dermal fibroblasts (P10858 HDFa from Innoprot, Derio, Spain) were seeded into 96-well plates (subculture level 3) at a density of 5 × 103 cells/well in 100 µL of fibroblast medium (FM). The incubation lasted 24 h in a temperature-regulated incubator at 37 °C, 95% humidity, and 5% CO_2_. The medium was removed after the incubation period and substituted with 100 µL of FM medium containing different concentrations of HA and HC. After that, they were distributed into six replicate wells and incubated with the above-mentioned conditions at different times. Controls consisted of untreated cells that were also distributed into six replicate wells and incubated using the same conditions as treated cells.

A preliminary viability assay was performed after 24 h of culture incubation with 25 different combinations of HC/HA concentrations (Table 1). The results of this preliminary assay were used to select 5 combinations (0, 0.125/0.5, 0.125/1, 0.25/0.5, and 0.25/0.5 mg/mL) for the viability, genetic expression, and collagen synthesis assays after 3, 6, 12, 24 h, 48 h, and 72 h of incubation. Cell viability was performed using the PrestoBlue™ Cell Viability Reagent (Invitrogen by Life Technologies, Carlsbad, CA, USA), following the manufacturer’s instructions. Plates were read in a Synergy MX Microplate Reader (Biotek, Winooski, VT, USA) at 600 nm (resazurin) and 570 nm (resorufin). Cell viability was normalized using the viability of the control wells for each incubation period.

#### 4.1.3. RNA Isolation and Quantitative Reverse Transcriptase Polymerase Chain Reaction (qRT-PCR)

Total RNA from each well was extracted with Cells-to-CT 1-Step TaqMan Kit (Thermofisher, Vilnius, Lithuania). Briefly, once the collagen hydrolysate medium was removed, cells were rinsed twice with 100 µL of cold phosphate buffer saline (PBS). After this, 50 µL of the lysis buffer provided with the kit was added to each well and mixed thoroughly with the cells, leaving the mixture to stand for 5 min at room temperature. Then, 10 µL of the stop solution was added to each well, mixed, and incubated for 2 min. Finally, RNA measurement was performed by fluorimetry using Qubit v3.0 fluorimeter (Life Technologies, Eugene, OR, USA) with the Qubit RNA HS assay kit. RNA extracts were stored at −80 °C until the RNA expression assays were performed.

Quantitative real-time reverse transcription polymerase chain reaction (qRT-PCR) was used to measure the COL-I gene expression. Expression of a housekeeping gene, glyceraldehyde 3-phosphate dehydrogenase (GAPDH), was used as a reference. The sequences of the primers and MGB probes used are as follows: GAPDH-F: 5′-GGAAGCTCACTGGCATGGC-3′, GAPDH-R: 5′-TAGACGGCAGGTCAGGTCCA-3′, GAPDH-P (probe): 5′-VIC-CCCCACTGCCAACGTGTCAGTG-MGB-3′, COL_I-F: 5′-ATGCCTGGTGAACGTGGT-3′, COL_I-R 5′-AGGAGAGCCATCAGCACCT-3′, COL_I-P (probe): 5′ 6-FAM-ACCAGCATCACCTCTGTC-MGB-3′. qRT-PCR assays were carried out on 7500 Fast Real-Time PCR System equipment (Applied Biosystems, Waltham, MA, USA) using the TaqMan^®^ 1-Step qRT-PCR Mix. Reactions were performed in a total volume of 20 µL in a MicroAmp fast optical 96-well reaction plate. Each reaction contained 10 ng of RNA, TaqMan^®^ 1-Step qRT-PCR Mix (1X), water, and a final concentration of 600 nM for each COL-I primer, 400 nM for each GAPDH primer, and 200 nM for both probes. The following thermal cycling protocol was applied: 50 °C for 5 min, 20 s at 95 °C, and then 40 cycles at 95 °C for 15 s followed by 1 min at 60 °C. qRT-PCR data were analyzed using the ΔΔCt method and normalized to the GAPDH values [30].

#### 4.1.4. Human Pro-Collagen I Quantification from Fibroblast Cell Culture Supernatants by Sandwich ELISA

Collagen hydrolysate medium was removed from polypropylene 96-well plates and centrifuged at 2000× *g* for 10 min at 4 °C. Supernatants were diluted at 1:20, 1:50, 1:200, 1:800, and 1:1600, depending on the cell incubation times of 3, 12, 24, 48, and 72 h, respectively. Then, the amount of pro-collagen I was determined by ELISA employing the kit “Human Pro-Collagen I α 1/OLIA1” (R&D Systems, Abingdon, UK), following the manufacturer’s instructions, including some modifications in the incubation conditions with the capture and detection antibody (1 h at 37 °C instead of 2 h at room temperature). Finally, plates were read using a spectrophotometer (Synergy Mx from Biotek Instruments, Winooski, VT, USA) at 450 nm with a wavelength correction set to 540 nm. For data analysis, the software “Four Parameter Logistic Curve” online data analysis tool was employed (https://www.myassays.com/four-parameterlogistic-curve.Assay (accessed on 17 October 2019)) [45].

### 4.2. Cosmetic Preparation

#### 4.2.1. Formulation

The final formulation of the cosmetic preparation was made using an oil-in-water base in which the oil phase was prepared with first-pressure almond oil, first-pressure Jojoba oil, emulsifier (Protelan ENS), and co-emulsifiers/stabilizers, such as Cetyl Alcohol and Cetyl palmitate. The aqueous phase was prepared with purified water, a thickener, and stabilizer obtained from algae combining carrageenan with glucose. It has also been added an acrylate as a formula stabilizer, a combination of sodium benzoate and potassium sorbate as a preservative system, tocopherol as an antioxidant to prevent rancidity of the oils, and lactic acid as a pH regulator. To this emulsion base, 5% (*w*/*w*) of active ingredient (AI) was added. The active ingredient was formulated with a combination of hydrolyzed collagen, glycogen, a hyaluronic acid mixture of both low and high Mw, and different commercial algae extracts together with different additives and preservatives in proportion; see Table 2.

#### 4.2.2. In Vitro Skin Irritation Assessment of Cosmetic Preparation according to RHE Test Method OECD 439

To perform the in vitro skin irritation test [22] of cosmetic preparation, 25 mg of product (at 100% concentration) was topically applied onto the surface of the RHE Model EpiDermTM epi-200-SIT for 60 min at 37 °C. At the end of the exposure period, tissues were rinsed with DPBS buffer and transferred to fresh medium for 24 h. Then, the medium was changed, and cells were incubated for an additional 18 h. Cell viability quantification was developed through MTT assay. Thus, tissues were treated for 3 h at 37 °C with 0.3 mL of MTT solution, and then 2 mL of Isopropanol was added and left for 2 h at room temperature with gentle agitation for formazan extraction. The concentration of formazan was measured by determining the absorbance at 570 nm on a scanning multiwall spectrophotometer (Halo Led 96, Dynamica Scientific Ltd., Livingston, UK).

### 4.3. Statistical Analysis

Statistical analysis was performed with IBM SPSS 28 software (IBM Corporation, Armonk, NY, USA). Regarding fibroblast cell viability, mRNA expression and pro-collagen I synthesis values were expressed as the mean value and standard deviation of six independent samples using one-way ANOVA followed by Scheffé test or by Welch ANOVA followed by Games–Howell when non-uniform variances were detected. To find out if there was an interaction between HC and HA, the ANOVA test of two factors was used. *p*-values < 0.05 were considered significant. To calculate the effect size (ES), eta squared (η2) was used for measuring the magnitude of a treatment effect. Correlations between every two variables were assessed using Pearson’s correlation coefficient (*p* values < 0.01). Regarding the in vitro skin irritation assessment of ingredients and cosmetic formulation, values were expressed as a mean value and standard deviation of five and four replicates, respectively. Data were statistically analyzed using one-way ANOVA test with statistical significance set at *p* < 0.05, followed by Dunnett’s multiple comparisons test.

## 5. Conclusions

This study demonstrates the potential valorization of fish-filleting skin by-products, mussel-processing by-products, and tuna viscera by-products for the recovery and bioproduction of three bioactive molecules: hydrolyzed collagen, glycogen, and hyaluronic acid, respectively, which could be safely employed as cosmetic ingredients. This study demonstrates the potential use of particular HC/HA combinations as stimulators of collagen I synthesis in fibroblast cultures. These findings also provide safety information regarding the potential use of those biomolecules in the formulation of a cosmetic preparation, concluding that both ingredients and cosmetic preparation were not irritants for the skin following the internationally validated reference method OECD 439 [22]. Further studies are needed to evaluate the cosmetic significance of the combination of these molecules on skin models and then in human volunteers.

## Figures and Tables

**Table 1:** Treatment number keys for the combinations of HC and HA concentrations employed in the study (a) and the corresponding cell viability values (%) for the fibroblast cell culture treated for 24 h with 25 different combinations of HC/HA concentrations (b).

**(a)**					
**HC (mg/mL)**	**HA (mg/mL)**				
	0	0.125	0.25	0.5	1
0	0	1	2	3	4
0.125	5	6	7	8	9
0.25	10	11	12	13	14
0.5	15	16	17	18	19
1	20	21	22	23	24
**(b)**					
**HC (mg/mL)**	**HA (mg/mL)**				
	0	0.125	0.25	0.5	1
0	100	82.60	78.79	78.43	74.85
0.125	95.36	86.92	86.15	83.91	66.68
0.25	87.18	84.86	82.26	85.24	72.49
0.5	90.28	89.13	77.25	74.96	59.43
1	87.01	87.59	86.25	81.64	64.22

**Table 2:** Components of the active ingredient used for the elaboration together with the emulsion base of the cosmetic preparation.

Components of the AI	% (*w*/*w*)
Water	46.13
Glycerin	28.2
Glycogen	10
Hydrolyzed collagen	8
Hyaluronic acid	3
Algae extract	3.75
Sodium benzoate	0.3
Potassium sobrate	0.2
Gluconolactone	0.18
Calcium gluconate	0.235

## Data Availability

The data presented in this study are available on request from the corresponding author.

## Supplementary Materials

The following supporting information can be downloaded at https://www.mdpi.com/article/10.3390/ijms242015380/s1.
