# Supplementary material for: Production and Partial Characterization of Bioactive Compounds from Underutilized Marine Bioresources for a Cosmetic Formulation: Cytotoxicity and Bioactivity Evaluation
Source: Int J Mol Sci. 2023 Oct 19;24(20):15380. doi: 10.3390/ijms242015380 (PMC10607788; doi:10.3390/ijms242015380)
Supplement: Supplementary file 1 [file ijms-24-15380-s001.zip › ijms-2622191-supplementary.pdf]

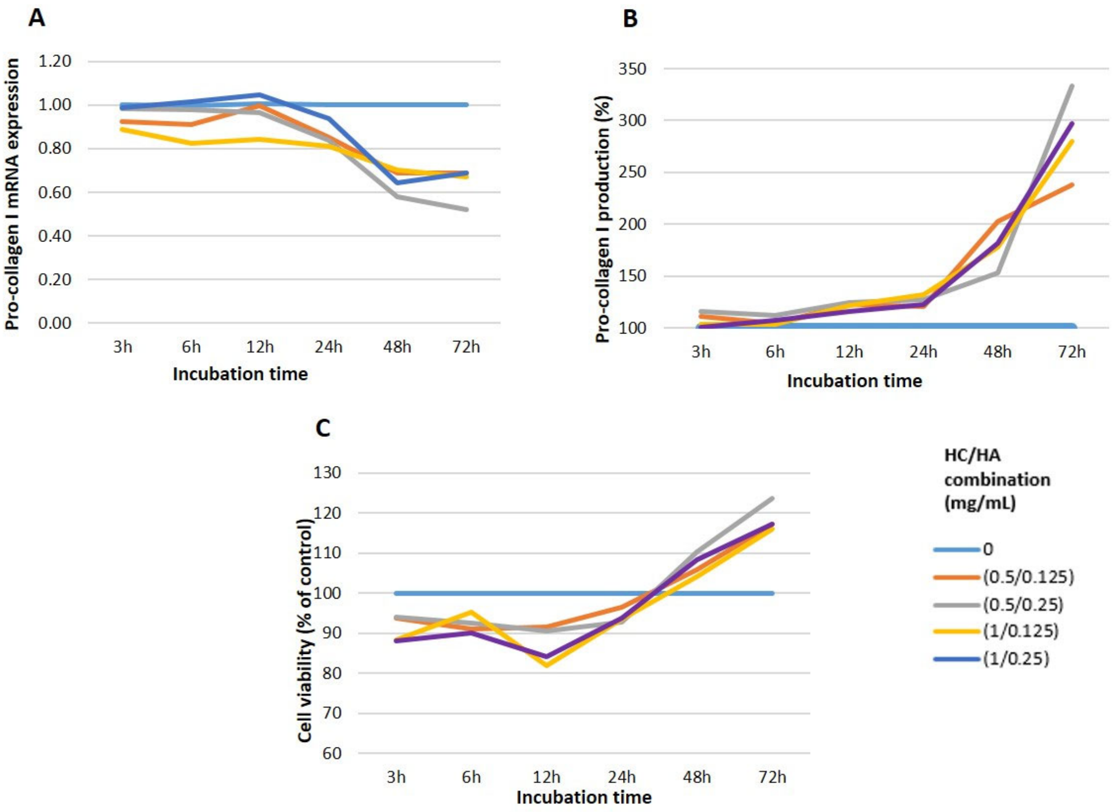

Figure S1. Procollagen I mRNA expression (A), Procollagen I syntesis (B) and cell viability (C) from 3 to 72 h of HC/HA treatment incubation.

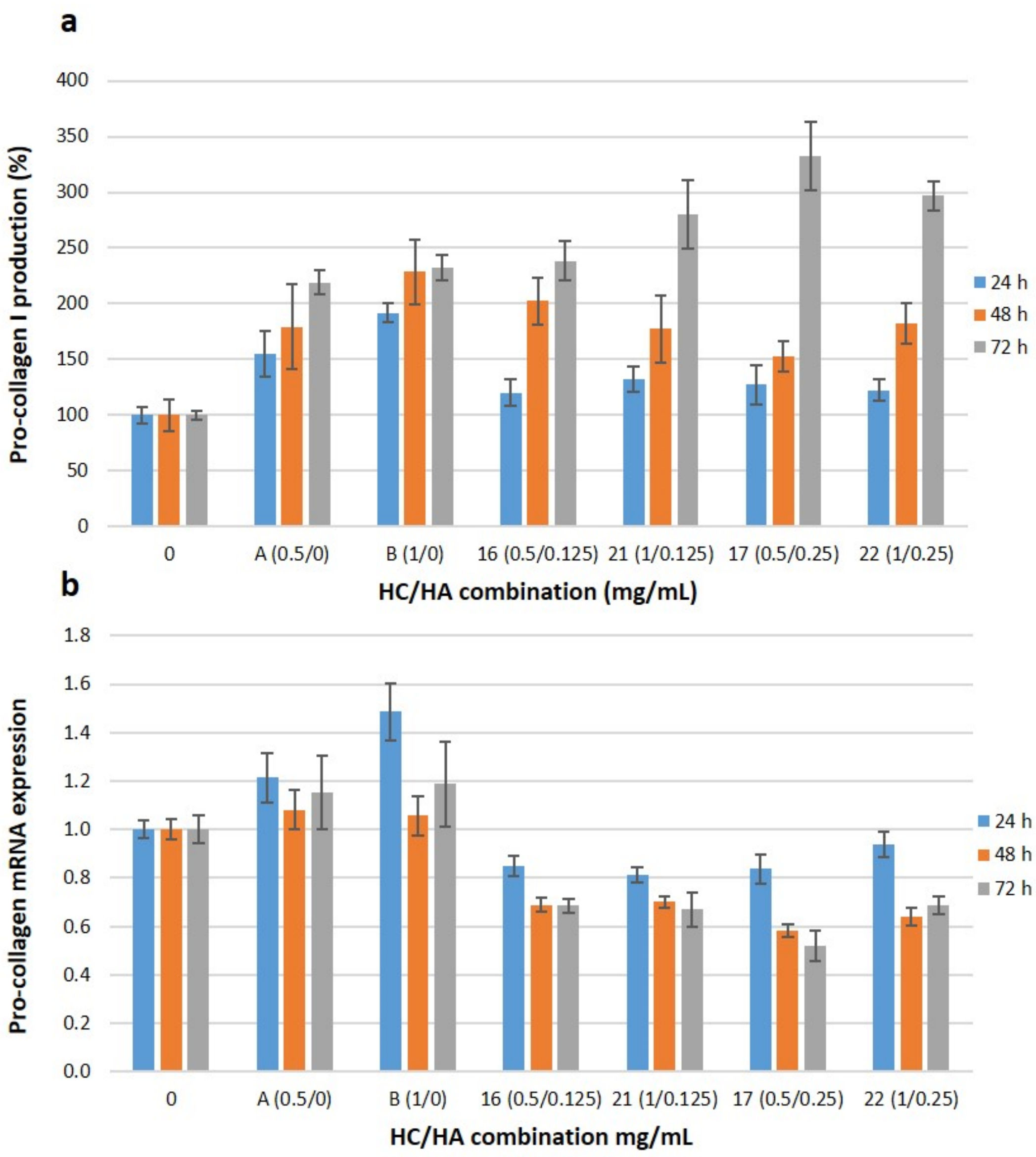

Figure S2. Pro-collagen I production (a) and Pro-collagen mRNA expression (b). A and B correspond to treatments used in Blanco et al.[11].
